# Suffocation

**Published:** 1828-02

**Authors:** 


### HOSPITAL REPORTS.

### SUFFOCATION.

### *Cases of Suffocation from the Respiration of the Fumes arising from burning Coals.* (Guy's Hospital.)

William Garbett, John Jackson, and John Harman, were brought into Guy's Hospital, at half-past nine on Saturday morning, Dec. 16th, in a state of insensibility. The circumstances which gave rise to the occurrence were as follow:—The above-mentioned men formed part of the crew of the Austria, of Whitby, and were in perfect health on Friday. At nine o'clock in the evening they retired, together with another of the crew, named Fairfoot, into the forecastle of the ship, for the purpose of going to rest. Before going to bed, the fire, which was contained in a grate placed in the centre of the room, was replenished with coals. At eight o'clock on the following morning, the usual signal was given by the mate for their appearance upon deck; but no answer being returned, he descended to learn the cause, and the following appearances presented themselves: Garbett and Fairfoot were lying on the floor, but Jackson and Harman remained in their beds; they were all in a state of stupor and insensibility, from which it was found impossible to rouse them, and they were brought up upon the deck, manifesting no other sign of life than a difficult and laborious breathing. The air of the forecastle was of a sulphurous and suffocating character, and, on investigation, it was found that the smoke and fumes arising from the combustion of the coals had been prevented from escaping by the removal of the upper division of the chimney, so that the orifice was several inches below the level of the deck, and consequently the smoke, instead of ascending, would be driven downwards by the wind. The hatchway, which was large enough to admit a full-sized man, had been inclosed throughout the night. The coals which had been used were from the Ouse, and seemed to be of a very inferior quality, containing rather a large proportion of sulphur.

From the foregoing account it would appear that the atmosphere of the forecastle must have undergone a gradual deterioration, by the admixture of sulphurous and carbonic acid gases, which were generated by the combustion of the coals. The effects which were produced upon the persons who respired this impure air, although uniform in some respects, yet differed very widely in severity. This variety, however, is not at all explicable from any difference in the concomitant circumstances; since, as the subjoined cases will show, the mildest and most severe cases took place under circumstances precisely similar.

Case I.—— Fairfoot, a young man of healthy temperament, was found lying upon the floor, on his elbows and knees: when brought upon deck, he was breathing with difficulty, and was quite insensible; after having been exposed, however, about two hours to the open air, he began to exhibit signs of consciousness, and at eleven o'clock he was able to speak. Excepting the exhibition of a small quantity of brandy, no remedial measures were had recourse to. His resuscitation was preceded by vomiting.

At two o'clock, he was able to walk to the hospital, and seemed to suffer no material inconvenience. He could give no account of the occurrence, and was unconscious of having left his bed.

Case II.—Wm. Garbett, a robust and muscular man, aged thirty, was placed in circumstances very analogous to the former, and was found beside him in a similar position. When brought into the hospital, at half-past nine, he was completely insensible; his breathing obstructed and stertorous; his extremities cold; his pupils, though not dilated, perfectly insensible to light; and his pulse frequent and weak.

Friction with heated vinegar was immediately had recourse to, and some ammonia was administered. At eleven o'clock, he inhaled a bladder of oxygen gas: this produced a transient brightening of the countenance, the colour of the face becoming slightly redder. No permanent amendment, however, was effected: he remained much in the same state, brandy being at intervals administered, till one o'clock, when his breathing became gradually shorter, and at half-past one he expired. About two hours before death, the pulse was ninety, regular, and rather full.

*Sectio cadaveris.*—This took place about two hours and a half after death. The appearance of the face was like that of a man who had died from strangulation. On opening the head, very few morbid appearances presented themselves. The veins of the dura mater seemed a little engorged, but those of the pia mater were perfectly natural. There was rather a preternatural abundance of blood in the sinuses, and slight serous effusion between the tunica arachnoides and pia mater; the veins of the corpora striata were very turgid. In other respects, the brain was completely healthy.

*Chest:* The pleura and pericardium presented nothing remarkable. On opening the latter, the right side of the heart seemed much distended, and both the auricle and ventricle were found to contain a large quantity of fluid blood. The lungs had a dark appearance, and there was very considerable congestion in them. The trachea contained a small quantity of mucus, and its internal surface was tinged in many places by a black carbonaceous deposit.

The subjects of the two remaining cases were sleeping in the same bed, and placed under exactly similar circumstances.

Case III.—John Orman, a ruddy and healthy looking youth, apparently about eighteen. At the time of his admission, he was labouring under the following symptoms:—His respiration was rather difficult, pulse quick, and he was completely insensible.

Ammonia was administered, and heated vinegar rubbed over the body and extremities. In a short time the breathing became easier, and his nose became sensible to the impression of stimuli. In about four hours his face had assumed a natural appearance, and consciousness began slowly to manifest itself. He went on improving, and at two o'clock was able to answer questions correctly, and complained only of pain in the back.

About three o'clock he was bled, and recovered with no further inconvenience.

Case IV.—The subject of this case was a boy about fourteen. When brought into the hospital, his symptoms were very severe: the extremities were cold, the pulse very frequent and weak, the respiration difficult and laborious, the pupil of the left side was dilated, and but very slightly affected by the admission of light; that of the right side, however, was natural.

He was immediately rubbed with hot vinegar, and some ammonia and brandy were administered, by means of a tube introduced into the œsophagus. At eleven o'clock he inhaled a bladder of oxygen gas: this was followed for a short time by stronger and deeper respirations, and there was a transient amendment in the expression of the countenance, and slight, temporary redness in the face.

At twelve o'clock, the muscles of the left side were observed to be rigid, and often affected by spasmodic twitchings; the right arm was in almost continual motion, and the mouth was occasionally drawn to the left side. The temperature of the left side was perceptibly higher than that of the right. There was an almost incessant motion of the eyes produced by the alternate action of the abductor and adductor muscles. The pulse were ranging from 140 to 160 in the minute, were always small and weak, often fluttering and indistinct.

At one o'clock, a sinapism was applied to the chest.

About an hour from this time, he was seen by Mr. Morgan, (under whose care the cases were admitted,) when, no amendment having taken place, the jugular vein was opened. Very little blood, however, could be obtained, and it was therefore thought proper to open a vein in the arm: the blood taken from both amounted to six ounces. The effect of this was to produce for a few minutes an increase in the sensibility of the affected iris, and to render the pulse for a few minutes rather fuller.

In a short time after this, ten ounces of blood were drawn from the right temple by cupping. The pulse again rose for a few minutes, and the pupil remained afterwards more sensible to light.

No obvious permanent amendment was produced by these measures: his respiration continued to be difficult, and his pulse soon became again fluttering and indistinct, and, when perceptible enough to be counted, they were from 150 to 160 in the minute. Brandy and ammonia were administered at intervals, and oxygen was frequently inhaled. The latter never failed to produce a temporary excitement, during which the eyes were opened, and the respiratory muscles called into increased exertion.

He gradually became worse, his powers evidently decreasing. At seven in the evening, his breathing was short and quick, his pulse fluttering and not to be counted, and a cold perspiration bedewed the surface. A stimulating enema was administered, and oxygen again inhaled.

He rallied a little about ten o'clock, but soon sunk into his former state, and about four o'clock on the following morning he expired.

*Sectio cadaveris.—Head:* The dura and pia mater deviated scarcely at all from the healthy appearance; the veins of the corpora striata were turgid with blood, and coagula of blood were found in both the internal carotid arteries. There was slight effusion of serum at the base of the brain; and, when a section was made of the brain, the bloody points were found to be preternaturally numerous.

*Chest:* The pleura pulmonalis and costalis were united rather extensively by strong adhesions, apparently of long standing. A large quantity of partially coagulated blood was found in the right side of the heart and pulmonary arteries; and the vasa vasorum were unusually distinct at the origins of the aorta and pulmonary artery.

The lungs presented nothing worthy of notice.

---

